# The KT Jeang Retrovirology prize 2016: Frank Kirchhoff

**DOI:** 10.1186/s12977-016-0286-5

**Published:** 2016-08-05

**Authors:** Retrovirology Editorial

**Affiliations:** BioMed Central, 236 Gray’s Inn Road, London, WC1X 8HB UK

 Frank Kirchhoff studied Biology at the Georg August-University in Göttingen and performed his diploma and PhD thesis at the German Primate Center under the supervision of Gerhard Hunsmann. The main goal of his thesis was the generation of a novel molecular clone of HIV-2 from an infected cell culture. After a challenging start involving the screening of millions of bacteriophage lambda plaques and the repeated isolation of only the 5′-half of the viral genome, he finally succeeded in cloning and characterizing the full-length infectious molecular clone of HIV-2 BEN [1, 2].

After graduation in 1991, Kirchhoff obtained a fellowship from the German Ministry of Health and joined Ronald C. Desrosiers’ group at the New England Regional Primate Research Center (Harvard Med. School, Southboro) for postdoctoral studies. Initially, he worked on the envelope glycoprotein of SIV and showed that the V3 loop region of SIVmac Env determines the viral coreceptor tropism [3] and functionally cooperates with the C4 region [4]. Kirchhoff was also part of a pioneering study showing that live-attenuated *nef*-deleted SIVmac239 efficiently protects rhesus macaques against challenge with pathogenic wild-type virus [5]. Initially, all his attempts to detect the vaccine or wild-type viruses in these animals by PCR failed, raising strong doubts about his ability to perform this “novel” technique. Later, it turned out that *nef*-deleted SIVmac strains lose large parts of their LTR (including some selected primer binding sites) because they serve mainly as *nef* coding sequences in vivo [6]. Another exciting finding also involved PCR: just before starting a new position in Germany, he obtained evidence for the long-sought after presence of defective *nef* genes in a long-term survivor of HIV-1 infection. Thus, after signing his contract in Germany he moved back to the US for a couple of months to characterize the first described long-term survivor infected by a *nef*-deleted HIV-1 strain [7].

In 1994, Kirchhoff established an independent research group at the Institute of Clinical and Molecular Virology (University of Erlangen) under the directorship of Bernhard Fleckenstein. He maintained an interest in Env function and HIV entry. Together with Stefan Pöhlmann, his first student as independent researcher (who became the successor of Kirchhoff`s first boss, Gerhard Hunsmann, ~15 years later), he analyzed the utilization of alternative coreceptors, such as BOB/GPR-15 and BONZO/STRL-33, by HIV-1 and SIVmac [8–11]. In cooperation with Robert Doms, he also examined the mechanisms underlying the interaction of HIV-1 with DC-SIGN and showed that virus binding and transfer are separable activities [12, 13]. Kirchhoff further continued collaborating with Jacek Skowronski, John L. Sullivan and Thomas C. Greenough to identify another long-term survivor with HIV-1 infection harboring defective *nef* alleles [14] and to define the mechanisms underlying CD4 and MHC-I down-modulation by HIV and SIV Nefs [15–18].

One of Kirchhoff`s main research interests was to elucidate the relative contribution of the many in vitro functions of Nef to viral replication and pathogenesis in vivo. To address this, he collaborated with Christiane Stahl-Hennig from the German Primate Research Center (Göttingen) to analyze the properties and evolution of specific mutant Nef SIVmac239 constructs in rhesus macaques. These studies provided evidence for the relevance of Nef-mediated modulation of CD4 [16, 17] and MHC-I [18, 19] as well as enhancement of viral infectivity and replication [16, 20] in vivo. However, they also showed that Nef’s interaction with the p21-activated kinase 2 (PAK2) is dispensable for AIDS progression in macaques [21, 22]. In cooperation with Leonid Margolis, he confirmed the importance of the HIV-1 accessory genes and Nef-mediated downmodulation of CD4 in ex vivo infected human lymphoid tissues [23, 24]. Together, these studies showed that Nef enhances viral replication and immune evasion in vivo by multiple genetically separable mechanisms and illustrated the enormous capacity of HIV-1 and SIVmac to “repair” attenuating point mutations in *nef* [25, 26] and to adapt to their respective host environment [27, 28].

Kirchhoff noted that most Nef functions are conserved between HIV-1, HIV-2 and SIV [29–34] and showed that the HIV-1 *nef* gene can largely substitute for the SIVmac *nef* in infected rhesus macaques [35]. However, in cooperation with Beatrice H. Hahn and others, he also identified some interesting differences. For example, HIV-1 Nefs are usually less active than those of most SIVs or HIV-2 in downmodulating CXCR4 [36] and CD28 [37]. Most interestingly, he found that the Nef proteins of HIV-1 and its *vpu* containing SIV precursors generally lack the TCR-CD3 downmodulation function and render infected T cells hyper-responsive to stimulation, whereas HIV-2 and most SIV Nefs suppress T cell activation and apoptosis [37–39]. Thus, it was tempting to speculate that this fundamental difference in Nef function might contribute to the chronic inflammation that drives progression to AIDS and is absent in some natural SIV infections [40]. In fact, inefficient downmodulation of TCR-CD3 by Nef (i.e. a more “HIV-1-like” phenotype) was associated with low numbers of CD4+ T cells in natural SIVsmm infection of sooty mangabeys [41] and in viremic HIV-2 infection [42]. Clearly, many host and viral factors play a role in the clinical outcome of HIV and SIV infection [43]. Nonetheless, his results support that the loss of Nef-mediated downmodulation of CD3 may have predisposed the retroviral lineage that resulted in the emergence of HIV-1 to higher pathogenicity.

Kirchhoff was curious to find out why the CD3 downmodulation function of Nef was specifically lost in primate lentiviruses encoding the viral *vpu* gene [37, 44]. Recent collaborative studies with Beatrice H. Hahn, Guido Silvestri and others provide first insights into the possible link between Vpu and Nef function. TCR-CD3 mediated T cell activation induces antiviral gene expression via NF-κB activation. Together with Daniel Sauter, a talented scientist in his lab, Kirchhoff showed that HIV-1 and its simian precursors utilize Vpu to prevent NF-κB activation [45]. Most other primate lentiviruses might achieve the same by Nef-mediated downmodulation of the TCR-CD3 receptor from the cell surface [37]. Notably, Nef proteins initially boost IKKβ-induced NF-κB activation irrespectively of their CD3 downmodulation function [45]. Thus, primate lentiviruses seem to use different strategies to boost NF-κB activation early during the viral life cycle to initiate proviral transcription and to suppress NF-κB-dependent antiviral gene expression at later stages. Finally, Kirchhoff observed that highly unusual CXCR4-tropic SIVsmm strains that cause almost complete loss of CD4+ T cells, lost their ability to downmodulate TCR-CD3 by evolving mutations disrupting the interaction of Nef with the CD3 ζ chain [46]. A possible explanation for this coevolution of Env and Nef function is that CD3 downmodulation may be advantageous for viral replication in activated CCR5 + memory T cells but not in naive CXCR4+ T cells. These studies thus provide insights into the interplay between different viral properties and may help to explain why HIV-1 more frequently switches to CXCR4 usage than SIV strains.

In 1998, Kirchhoff met Wolf-Georg Forssmann, who pioneered the isolation of endogenous bioactive peptides from complex libraries generated from human hemofiltrate. This source becomes available in thousands of liters from patients with renal failure and contains all peptides circulating in human blood. They decided to cooperate and a highly talented and tenacious diploma student (Jan Münch, who has meanwhile gained a full professorship in Ulm but lost his Rasta curls) started his work on the discovery of novel inhibitors of HIV infection. The initial studies identified a 20-residue C-proximal fragment of α1-antitrypsin, designated VIRus-Inhibitory Peptide (VIRIP) that inhibits HIV-1 infection by specific interaction with the viral gp41 fusion peptide [47]. An optimized derivative of this peptide was safe and effective in a phase I clinical trial [48] and current interdisciplinary research efforts aim at making it more active and orally available. Perhaps most strikingly, Kirchhoff and collaborators isolated a peptide from human semen forming amyloid fibrils that strongly enhance HIV infection [49]. Subsequent cooperative studies with Nadia Roan and Warner Greene showed that cleavage products of at least two abundant seminal proteins (prostatic acid phosphatase and semenogelin) form amyloid fibrils that boost HIV infection [50, 51] and are found in semen from healthy individuals [52]. They further showed that the enhancing effect of these amyloids depends on their positive charge [53] and characterized effective inhibitors [54, 55]. Recently, Münch and Kirchhoff also found that semen impairs the antiviral efficacy of microbicides [56] possibly explaining their poor efficacy in clinical trials. While the relevance of seminal amyloids for HIV transmission in vivo is still largely unclear [57], they even provided a basis for the development of nanofibrils as potent enhancers of retroviral gene transfer [58]. Thus, the strategy of utilizing peptide libraries from body fluids generated some unexpected and exciting results [59]. This is still continuing, e.g. with the recent discovery of a small fragment of serum albumin (EPI-X4) as an effective and highly specific CXCR4 antagonist that may represent a key regulator of CXCR4 signaling and an inhibitor of CXCR4-tropic HIV-1 strains in vivo [60].

Another key interest of Kirchhoff is the role of viral properties in the spread of HIV/AIDS. In cooperation with last year`s awardee, Paul Bieniasz, he showed that most SIVs use Nef to counteract the restriction factor tetherin [61]. However, Kirchhoff also found that the human tetherin ortholog is resistant to Nef due to a deletion in its cytoplasmic domain and that only the Vpu protein of pandemic HIV-1 group M evolved full activity against human tetherin [62]. In contrast, Vpus from rare group N viruses acquired only modest anti-tetherin activity and lost the CD4 degradation function [62] but are still evolving towards higher activity against tetherin [63]. Kirchhoff found that HIV-1 group P and O Vpus are inactive against human tetherin [64]. Recently, however, he noted that O-Nefs evolved the ability to target a different region in the cytoplasmic part to counteract human tetherin which helps to explain the epidemic spread of this group [65]. Kirchhoff also showed that HIV-1 can rapidly reacquire Nef-mediated anti-tetherin activity in its original chimpanzee host [66] and that tetherin antagonism significantly contributes to replication fitness and interferon resistance of transmitted-founder HIV-1 strains in CD4+ T cells [67]. Together with Daniel Sauter, he further demonstrated that the antiviral activity of tetherin [68] and the protective deletion in the human ortholog [69] have an ancient origin. Altogether, these data illustrate the importance of tetherin antagonism in the evolution and spread of HIV/AIDS. Finally, Kirchhoff is interested in the discovery of novel restriction factors. He joined forces with Amalio Telenti to perform genome-wide screens for cellular genes sharing features of those encoding known antiviral restriction factors [70]. Detailed characterization of one of them showed that guanylate binding protein 5 (GBP5) inhibits HIV-1 by interfering with Env function and can partly be evaded by increasing Env expression at the cost of Vpu function [71].

Kirchhoff received several awards including the Gottfried Wilhelm Leibniz award of the DFG (2009) and an ERC Advanced investigator grant (2012). He is currently an editor of the Journal of Virology and serves on various editorial and advisory boards. In 2012, Kirchhoff became cofounder of the competence center Ulm Peptide Pharmaceuticals (U-PEP). Besides his passion for table tennis that he is indulging daily after lunch for at least 1 h, he is highly interested in innovative interdisciplinary research and is co-applicant and one of four founding directors of Ulm University’s Centre of Quantum Biological Science (ZQB). In 2009, he was elected member of the German Academy of Sciences Leopoldina.
