# Erratum to: The KT Jeang Retrovirology prize 2016: Frank Kirchhoff

**DOI:** 10.1186/s12977-016-0303-8

**Published:** 2016-09-16

**Authors:** Retrovirology Editorial

**Affiliations:** BioMed Central, 236 Gray’s Inn Road, London, WC1X 8HB UK

## Erratum to: Retrovirology (2016) 13:53 DOI 10.1186/s12977-016-0286-5

Unfortunately, the original version of this article [1] contained an error. Figure 1 was not included. Figure 1 has been included in the original article and is also included correctly below.

**Fig. 1:**
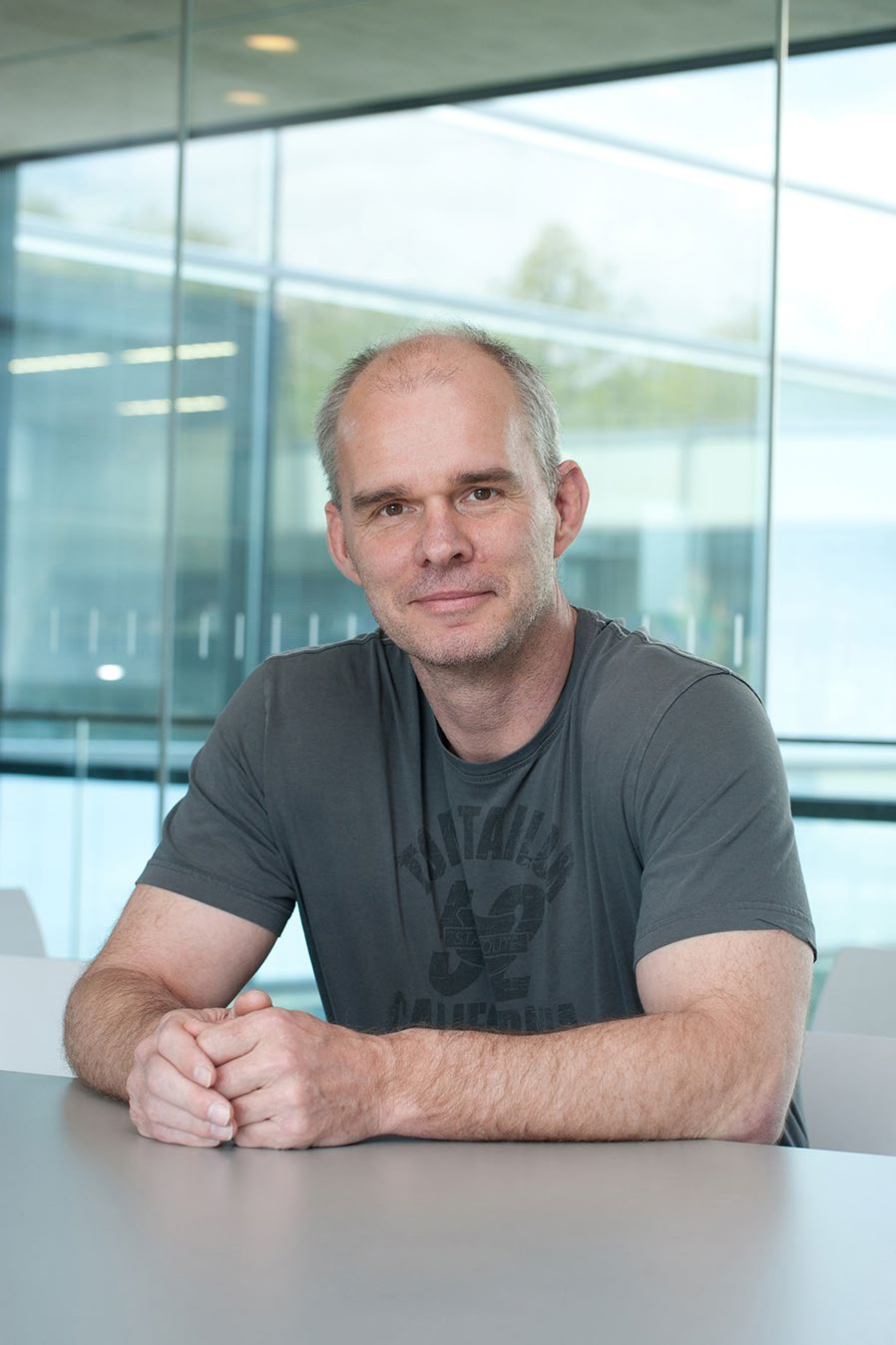
Frank Kirchhoff
